# Comparison of Tracheal Intubation Using the Air-Q ILA and LMA Blockbuster Among Adults Undergoing Elective Surgery: A Randomized Controlled Trial

**DOI:** 10.4274/TJAR.2024.241624

**Published:** 2024-09-17

**Authors:** Kavitha Girish, Thilaka Muthiah, Dalim Kumar Baidya, Renu Sinha, Vimi Rewari, Souvik Maitra, Manpreet Kaur, Rajeshwari Subramaniam

**Affiliations:** 1 Baby Memorial Hospital, Clinic of Anaesthesiology, Kozhikode, India; 2 Apollo Simulation Centre, Apollo Hospitals, Chennai, India; 3 All India Institute of Medical Sciences, Department of Anaesthesiology, Critical Care and Pain Medicine, Guwahati, India; 4 All India Institute of Medical Sciences, Department of Anaesthesiology, Pain Medicine and Critical Care, New Delhi, India; 5 Penn State Hershey Medical Center and Penn State College of Medicine, Department of Anaesthesia and Perioperative Medicine, Hershey, Pennsylvania

**Keywords:** Airway management, bronchoscopy, endotracheal intubation, glottis, laryngeal mask airway

## Abstract

**Objective:**

Air-Q intubating laryngeal airway (ILA) is associated with a 58-77% success rate in blind intubation. The newer laryngeal mask airway (LMA) blockbuster is specially designed to facilitate easier endotracheal intubation and may have a higher success rate. The current study aimed to compare the success rate of endotracheal intubation using the Air-Q ILA and LMA blockbuster.

**Methods:**

After ethics committee approval and informed written consent, 140 adult patients with normal airways who were scheduled for elective surgery under general anaesthesia requiring endotracheal intubation were recruited for this randomized controlled trial. Blind endotracheal intubation was performed using the Air-Q ILA in group A and the LMA blockbuster in group B with special maneuvers and/or tubes in the second attempt. Fibreoptic bronchoscope (FOB) guidance was used in the third attempt if required. The primary outcome was the success rate of intubation without FOB assistance. The number of attempts for supraglottic airway (SGA) insertion, the time taken for SGA insertion, and the overall intubation time was also noted.

**Results:**

The success rate of intubation without FOB guidance was significantly higher in group B than in group A [91.4% vs 55.7%; relative risk (RR) 1.68; (95% confidence interval (CI) 1.34, 2.11); p<0.0001]. The number of attempts for SGA insertion was similar in groups A and group B [87% vs 90%; RR 1.03; (95% CI-0.92, 1.16); p=0.60]. The times for successful SGA insertion and endotracheal intubation were also similar between the groups.

**Conclusion:**

The LMA blockbuster offers a significantly higher success rate for endotracheal intubation without FOB guidance than the Air-Q ILA in adult patients with normal airways. However, an increased success rate was achieved with the use of a specially designed flexible endotracheal tube and maneuvers.

**Main Points**

• Laryngeal mask airway blockbuster has a significantly higher success rate for blind endotracheal intubation than Air-Q intubating laryngeal airway in adult patients with a normal airway. However, the fiberoptic bronchoscopic glottic view, intubation time, and incidence of sore throat are similar.

## Introduction

Supraglottic airway (SGA) devices can be used as primary airway devices for ventilation or as conduits for intubation. It also provides a route for oxygenation in failed intubation, and those with a gastric port can facilitate the drainage of gastric contents.^1^ In difficult airways, a fibreoptic bronchoscope (FOB) may be considered the gold standard for intubation if available, and intubation through SGA can be a reasonable alternative in appropriate cases.^2^ Although intubation through SGA should be ideally guided by FOB, in a resource-limited setting, this approach may need to be performed without FOB assistance. Therefore, an SGA with a high blind intubation success rate is clinically significant.

Laryngeal mask airway (LMA) blockbuster, a novel SGA, has a short airway tube that matches the oropharyngeal curve, a guidance device at the laryngeal end that directs the endotracheal tube (ETT) toward the glottis, and comes with its designated wire-reinforced ETT with a soft tip that facilitates intubation. It has been found to have a 90-94% intubation success rate in initial studies compared with the 66% success rate of LMA Fastrach.^3, 4^ Air-Q intubating laryngeal airway (ILA) has a shorter and wider airway tube, has a removable connector, and comes with a stabilizing stylet to facilitate intubation. However, the intubation success rate through the Air-Q ILA has been 58-77% in comparative trials with the LMA Fastrach.^5, 6, 7^

The initial promise of LMA blockbuster was derived from smaller studies, and intubation success between LMA blockbuster and Air-Q ILAs was not compared directly. Therefore, this study compared the LMA blockbuster and Air-Q ILAs. The primary outcome was the success rate of blind intubation without FOB assistance, and the secondary outcomes were i) time to intubation, ii) success rate of SGA insertion, iii) FOB glottic view, and side effects like sore throat and blood on SGA removal. We hypothesized that the LMA blockbuster will have a higher success rate for blind intubation due to specific modifications to aid intubation and the availability of a specially designated ETT compared with the Air-Q ILA.

## Methods

This single-center, randomized, controlled trial was conducted at a tertiary care academic institution in India from October 2019 to April 2021. Permission was obtained from the Institutional Ethics Committee of All India Institute of Medical Sciences (ref no.: IECPG-446/27.06.2019), and the protocol was registered in a publicly accessible clinical trial registry database of India (www.ctri.nic.in; CTRI/2019/10/021623) before the recruitment of the first patient. Written informed consent was obtained from each patient.

### Study Population

One hundred and forty adult patients aged between 18 and 75 years with either sex and American Society of Anesthesiologists physical status I or II, who underwent elective surgery under general anaesthesia requiring endotracheal intubation, were recruited in this study. Patients with respiratory or pharyngeal pathologies, cervical spine disease, potentially difficult airway and patients at risk of regurgitation were excluded from the study.

### Randomization and Blinding

Patients were randomized according to a software-generated random number table in the two study groups. In group A, the Air-Q ILAs and in group B, the LMA blockbuster was used for securing the airway and as a conduit to intubation. Allocation was concealed using the sealed envelope technique. The anaesthesia team in the operating room opened the envelope and followed the previously mentioned procedure. The independent investigator noted all outcome data. Complete blinding was not possible for obvious reasons. The operator who performed SGA placement and subsequent intubation was blinded to the FOB view. The postoperative outcome assessor was also blinded to group allocation.

### Anaesthesia Protocol

All patients underwent a routine pre-anaesthesia assessment and were kept nil per oral for 8 hours for solids and 2 hours for water before surgery. In the operating room, an intravenous line was started, and routine monitoring, i.e. electrocardiography, pulse oximeter, non-invasive blood pressure, and capnography was performed. Patients were pre-oxygenated for 3 min, and anaesthesia was induced using intravenous Fentanyl (2 µg kg^-1^) and Propofol (2.0-2.5 mg kg^-1^). Neuromuscular blockade was achieved with atracurium at 0.5 mg kg^-1^, and after 3 min, a randomly assigned SGA was inserted.

In group A, the appropriate size of Air-Q ILAs was chosen according to the patient’s weight [size 2.5 (30-50 kg), size 3.5 (50-70 kg), size 4.5 (70-100 kg)]. Cuffed polyvinyl chloride (PVC) ETTs of sizes 6.5, 7.0, and 8.0 were selected for Air-Q ILA 2.5, 3.5, and 4.5, respectively. The ETTs were pre-warmed and lubricated with 2% lignocaine jelly. An Air-Q ILA was inserted using an inward and downward pressure using the curvature as a guide until resistance was felt, and the cuff was inflated to a cuff pressure of 60 cm H_2_O using a cuff pressure manometer.

In group B, size 3 LMA Blockbuster was used in patients weighing 30-50 kg, size 4 for 50-70 kg and size 5 for 70-100 kg. Cuffed PVC ETTs of sizes 6.5, 7.0, and 7.5 were selected for LMA Blockbusters of sizes 3, 4, and 5, respectively. The ETTs were pre-warmed and lubricated with 2% lignocaine jelly. The LMA blockbuster was directed into the pharynx using the curvature until resistance was encountered. The four-way connector was held with both thumbs, and the LMA Blockbuster was moved up and down to achieve adequate ventilation.

If the SGA was not successfully placed in the first attempt, the index finger of the left hand was placed behind the mask and flexed forward to guide the SGA into the pharynx. A mandibular lift was used for the third attempt to facilitate SGA insertion. If the SGA was not successfully placed in three attempts, the trachea was intubated using direct laryngoscopy. SGA placement was confirmed by observing an adequate chest rise and the appearance of square wave capnography. After confirmation of SGA placement, a FOB was performed to note the glottic view. Thereafter, the connector was removed, and the previously lubricated ETT was inserted into the airway tube, which was gently advanced further to intubate the trachea. The position was confirmed by auscultation and capnography. If the first attempt was unsuccessful, the ETT was withdrawn from the airway tube, and the manufacturer’s recommendations were used in the second attempt. In group A, the Air-Q ILA was withdrawn 5-8 cm and reinserted with a mandibular lift, and a bougie was inserted into the airway tube with the coude tip facing upwards.^6^ External laryngeal manipulation was performed to guide the bougie into the larynx if required, and subsequently, the lubricated PVC ETT was advanced over the bougie. For the second attempt in group B, the dedicated flexible ETT provided with the LMA Blockbuster was used after lubrication with lignocaine jelly, external laryngeal manipulation, and rotation of the ETT, if required. If intubation was unsuccessful after the second attempt in both groups, FOB guidance was used for the third attempt. If the third attempt was also unsuccessful, the trachea was intubated using a direct laryngoscope.

Anaesthesia trainees who had previously performed at least 50 similar procedures performed SGA placement and subsequent intubation. Patients were ventilated with 100% oxygen between attempts if necessary. If SpO_2_ decreased to 90% or less during the procedure, direct laryngoscopy and intubation were performed. Subsequent anaesthesia management was performed at the discretion of the anaesthesia team.

### Data Collection

Baseline and demographic data at enrollment and outcome data were recorded. The primary outcome was blind intubation success rate without FOB assistance (combined success rate of first and second attempt), and the secondary outcomes were;

**i)** Success rate of SGA placement,

**ii)** SGA insertion time: from the time the device entered the mouth until the appearance of the capnograph waveform. If no carbon dioxide was detected or the seal was inadequate, the device was removed. The time of the second/third attempt was recorded similarly, and the insertion time was considered the sum of all attempts.

**iii)** ETT insertion time: from the time of insertion of the ETT through the SGA until the appearance of the capnograph waveform. If no carbon dioxide was detected, the ETT was removed. The time of the second/third attempt was recorded similarly, and the insertion time was considered the sum of all attempts.

**iv)** Time for removal of SGA and blood on SGA: The SGA was removed after confirmation of successful intubation. The time needed to remove the SGA was recorded as the time from the initial disconnection of the ETT from the breathing circuit until reconnection and verification of the capnography waveform. Upon removal of the SGA, a note was made for any visible blood on the device, which indicated trauma to the upper airway.

**v)** Glottic view under FOB guidance: This was classified as per Brimacombe et al. into four grades: Grade 1: a globtic aperture seen completely without any obstruction, Grade 2: a globtic aperture seen partially but visual obstruction <50%, Grade 3: Glottic aperture barely seen and visual obstruction >50%, and Grade 4: a globtic aperture invisible.^8^ Grades 1 and 2 were considered favorable.

**vi)** An adverse airway event was defined as an oxygen desaturation of 90% or less, significant airway trauma, or other major adverse event.

**vii)** The incidence of postoperative sore throat (POST) was assessed 0, 1, 6, and 24 hours after surgery.

### Sample Size Estimation and Statistical Analysis

Based on previous studies, we assumed a success rate of intubation of 58% with Air-Q and 90% with Blockbuster LMA.^5, 9^ Considering the 80% power of the study and type 1 error as 0.05, 62 patients were required in each group. However, anticipating a dropout rate of 10%, we included 70 patients in each group.

## Results

**Figure 1:**
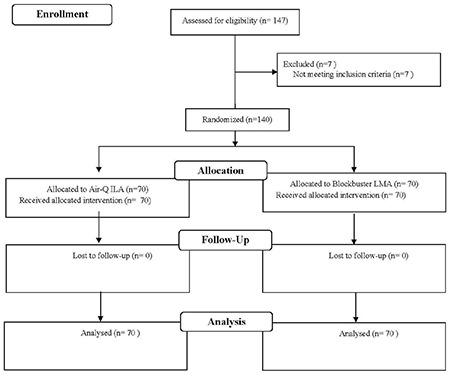
CONSORT flow diagram ILA, intubating laryngeal airway; LMA, laryngeal mask airway

In total, 140 patients were recruited and analyzed (Figure 1). Demographic and baseline data were comparable between the groups (Table 1). Successful unassisted intubation was possible in 64 out of 70 patients in group B (91.4%) and 39 out of 70 patients in group A (55.7%) (*P* < 0.001). The overall intubation success rate was 100% in both groups. SGA placement success rates were similar between the groups (Table 2). Only 3 patients in group A and 1 patient in group B required a third attempt for SGA insertion.

The time to SGA insertion and intubation were comparable between both groups (Table 2). A favorable glottic view (combination of grades 1 and 2) was observed in 81% in the Air-Q ILA group and 77% in the LMA Blockbuster group (*P*=0.53).

The presence of blood on the SGA was observed in 24 patients on Air-Q ILA and 15 patients on LMA Blockbuster, and the incidence of POST at all time points was comparable between the groups (Table 3).

## Discussion

In the present study, we observed a higher intubation success rate without FOB assistance in the LMA Blockbuster group compared with the Air-Q ILA. However, the first attempt intubation success rate, SGA insertion success rate, favorable FOB glottic view, intubation time, and incidence of sore throat were similar.

In the present study, the success rate of blind intubation was significantly better with the LMA Blockbuster (91.4%) compared with the Air-Q ILA (55.7%) [relative risk- 1.64; (95% confidence interval) (1.31, 204); *P *< 0.0001]. This is similar to the reported high success rate of blind intubation through LMA Blockbuster by Endigeri et al.^3^ (90%) and Singh^4^ (94%). However, the success rate of blind intubation with Air-Q ILA was 55.7% only, and FOB guidance was required in the remaining 44.3% of patients. This result is similar to the pilot study by Bakker et al.^5^ where only a 58% success rate was achieved for blind intubation in the first attempt. They attributed the low success rate to the absence of an ETT with Air-Q ILA and the learning curve. Karim and Swanson^6^ observed a 77% success rate for blind intubation in two attempts with the Air-Q ILA and 99% with the LMA Fastrach. They attributed the better success rate of intubation with the LMA Fastrach to the specialized tube available as compared to the standard ETT with the Air-Q ILA. Similar to Karim and Swanson^6^, we have used special manoeuver and bougie guidance for intubation through AirQ-ILA in the second attempt, which marginally improved the success rate compared with the first attempt.

It is interesting to note that the first attempt success rate was similar in both groups when we used a PVC ETT tube, and the success rate improved markedly in the LMA Blockbuster after the use of the specially designed ETT. Mohan et al.^9^ reported success rates of 84% and 96% with PVC ETT and specially designed ETT through LMA Blockbuster. However, in view of such improvements in success rates in both groups in the second attempt, though marginal in Air-Q ILA and marked in LMA Blockbuster, we suggest that a specially designed ETT with rotating/twisting movements during advancement should be used in LMA Blockbuster,^10^ and bougie guidance with a described maneuver should be used in Air-Q ILA in the first attempt itself for the best results if blind intubation is attempted.^6^

Moreover, the unavailability of an appropriate Air-Q ILA size could have contributed to a lower intubation success rate through the latter. In our experience, size 3.5 seemed to be too large and size 2.5 too small for some patients, especially females weighing around 50 kg. This could lead to misalignment and subsequent intubation difficulties.

In the present study, all patients were successfully intubated in the final FOB guided attempt. Similarly, Karim and Swanson^6^ reported a 96.7% success rate in FOB-guided intubation using air-Q ILA. Samir and Sakr^11^ also reported a 96.7% success rate in FOB-guided intubation using Air-Q ILA in patients with limited cervical spine instability. El-Ganzouri et al.^12^ found blind intubation success rates of 70% and FOB-guided intubation success rates of 97.5% through Air-Q ILAs with a shorter insertion time. Both SGA devices should be considered useful for FOB-guided endotracheal intubation because it was finally possible to intubate all cases using the FOB.

Although favorable glottic views were similar between the groups (81% in Air-Q ILA and 77% in LMA Blockbuster), grade 1 glottic views were observed in 70% of the Air-Q cases and only 45% of the LMA Blockbusters cases. Despite the comparatively poor glottic view, the LMA Blockbuster achieved a higher success rate for blind intubation. Similarly, Endigeri et al.^3^ observed full FOB view of the glottis in 43% of cases, partial glottic view in 30%, and only epiglottis in 20%, but achieved a 90% blind intubation success rate. This suggests that LMA Blockbuster may facilitate successful intubation despite the poor glottic view. A multitude of factors, including a short airway tube with >95º angulation which aids exit of ETT at 30º acute angle from the laryngeal mask with the help of a guidance ramp at the laryngeal end, and specially designed soft-tip ETT with rotation movement may facilitate blind intubation through LMA Blockbuster.^13^

In the present study, the first-pass insertion success rates were 87% for the Air-Q ILAs and 90% for the LMA blockbusters. This is very similar to previous studies. Neoh and Choy^7^ observed a 96% success rate, Galgon et al.^14^ reported an 88% success rate in the first attempt for Air-Q ILA insertion, whereas Endigeri et al.^3^ found a 90% success rate with Blockbuster LMA.

The mean times to successful SGA insertion in the first attempt were 29 s in the Air-Q ILA and 27 s in the LMA blockbuster. Galgon et al.^14^ observed that the mean insertion time for the Air-Q ILA was 20 s, and in the study by Karim and Swanson^6^, the mean insertion time for the Air-Q ILA was 27 sec. Endigeri et al.^3^ reported a mean insertion time of 12 s for the LMA Blockbuster. The overall mean time to SGA insertion was 37 s in the Air-Q ILA group and 33 s in the LMA Blockbuster group.

In the current study, the median cumulative time for ETT insertion was 43 s in the Air-Q ILA and 31 s in the LMA Blockbuster. This was statistically not significant; however, could be clinically meaningful. A similar trend was observed in previous studies. The mean time for intubation using the LMA Blockbuster was 18 s according to the study by Endigeri et al.^3^. Karim and Swanson^6^ reported a mean intubation time of 35 s using Air-Q ILA.

The incidence of POST was 41% and 27% in the Air-Q ILA and LMA blockbuster groups, respectively, 1 h after surgery, and it reduced to 4% in both groups at 24 h. Neoh and Choy^7^ reported 51% sore throat with Air-Q ILA. The increased incidence of POST could be attributed to multiple attempts in the Air-Q ILA group and the use of PVC tubes in the first attempt in the LMA Blockbuster group.

The important strengths of the present study include a larger sample size than that of previously published literature; complete follow-up with no data loss and a more pragmatic design with the use of commonly available PVC ETT and assessment of blind intubation success rate considering the relevance and wider application of this technique in resource-poor settings where difficult airway patients may need to be managed without FOB.

### Study Limitations

The limitation of our study could be the lack of inclusion of obese patients and other patients with difficult airway, where the study could be more relevant. The current findings need to be validated in those scenarios in further randomized controlled trials.

## Conclusion

LMA Blockbuster offers a significantly higher success rate of endotracheal intubation without FOB guidance than Air-Q ILA in adult patients with normal airways. However, an increased success rate in the LMA Blockbuster was achieved with the use of a specially designed, dedicated flexible ETT and external maneuvers.

## Ethics

**Ethics Committee Approval:** Permission was obtained from the Institutional Ethics Committee of All India Institute of Medical Sciences (ref no.: IECPG-446/27.06.2019), and the protocol was registered in a publicly accessible clinical trial registry database of India (www.ctri.nic.in; CTRI/2019/10/021623) before the recruitment of the first patient.

**Informed Consent:** Written informed consent was obtained from each patient.

## Figures and Tables

**Table 1. Patient Demographics; Mean (SD):** 

**Group**	**Air-Q ILA (n=70)**	**LMA Blockbuster (n=70)**	**Significance**
Age (years)	40.37±14	40.31±14	0.981
Female (%)	41 (58.6)	49 (70)	0.108
Weight (kg)	61 (9)	57(8)	0.032
BMI (kg m^-2^)	22.9±2	22.4±2	0.255
Height (cm)	163±10	160±9	0.108

Data expressed as mean±SD or number (percentage)

SD, standard deviation; ILA, intubating laryngeal airway; LMA, laryngeal mask airway; BMI, body mass index.

**Table 2. Number of Attempts and Time to SGA Insertion and Intubation in the Groups Air-Q ILA and Blockbuster LMA. Values are Presented as Median (IQR):** 

			**Air-Q ILA(n=70)**	**LMA Blockbuster** **(n=70)**	**Significance**
Success rate of blind intubation Intubation success in 1^st^ attempt Intubation success in 2^nd^ attempt Intubation success in 3^rd^ attempt (FOB guided intubation)			39 (55.7%) 33 (47.1%) 6 (8.6%) 31 (44.3%)	64 (91.4%) 39 (55.7%) 25 (35.7%) 6 (8.6%)	0.0001
Time to intubation (seconds)			43 (20-122)	31.5 (23-45)	0.122
Success rate of SGA placement		1^st^ attempt	61 (87.1%)	63 (90%)	0.759
		2^nd^ attempt	6 (8.6%)	6 (8.6%)	
		3^rd^ attempt	3 (4.3%)	1 (1.4%)	
SGA insertion time (seconds)			29 (22-35)	27 (21-32)	0.198
SGA removal time (seconds)			38 (30-43)	35 (30-41)	0.341
Glottic view grading (8)	Grade 1		49 (70%)	32 (45.7%)	0.004
	Grade 2		8 (11.4%)	22 (31.4%)	
	Grade 3		7 (10%)	13 (18.6%)	
	Grade 4		6 (8.6%)	3 (4.3%)	

Data expressed as frequency (percentage) or median (IQR)

SGA, supraglottic airway; ILA, intubating laryngeal airway; LMA, laryngeal mask airway; IQR, interquartile range; FOB, fibreoptic bronchoscope.

**Table 3. Blood on the SGA and Postoperative Sore Throat:** 

		**Air-Q ILAs (n=70)**	**LMA Blockbuster (n=70)**	**Significance**
Blood in the SGA		24 (34%)	15 (21%)	0.065
Sore throat	1 h	29 (41%)	19 (27%)	0.054
	6 h	13 (18.5%)	8 (11%)	0.172
	12 h	4 (6%)	3 (4%)	0.50
	24 h	3 (4%)	3 (4%)	0.660

Data expressed as number (percentage)

SGA, supraglottic airway; ILA, intubating laryngeal airway; LMA, laryngeal mask airway
